# OGUMI—A new mobile application to conduct common-pool resource experiments in continuous time

**DOI:** 10.1371/journal.pone.0178951

**Published:** 2017-06-05

**Authors:** Gunnar Brandt, Micaela M. Kulesz, Dennis Nissen, Agostino Merico

**Affiliations:** 1 Systems Ecology Group, Leibniz Centre for Tropical Marine Research, Fahrenheitstraße 6, 28359 Bremen, Germany; 2 Institutional & Behavioural Economics Group, Leibniz Centre for Tropical Marine Research, Fahrenheitstraße 6, 28359 Bremen, Germany; 3 Naymspace, Krusenrotter Weg 65, 24113 Kiel, Germany; 4 Faculty of Physics & Earth Sciences, Jacobs University Bremen, Campus Ring 1, 28759 Bremen, Germany; Middlesex University, UNITED KINGDOM

## Abstract

OGUMI is an Android-based open source mobile application for conducting Common-Pool Resource Experiments, Choice Experiments, and Questionnaires in the field, in the laboratory, and online. A main feature of OGUMI is its capacity to capture real-time changes in human behaviour in response to a dynamically varying resource. OGUMI is simple (for example, likewise other existing software, it does not require expertise in behavioural game theory), stable, and extremely flexible with respect to the user-resource model running in the background. Here we present the motivation for the development of OGUMI and we discuss its main features with an example application.

## Introduction

Common-Pool Resource Experiments (CPREs) are an integral part of the economists’ toolbox for analysing user harvest behaviours. Since the first CPRE—25 years ago [1]—a growing number of these experiments have been conducted for many different resources, using different sample populations, and with participants of vastly diverse backgrounds, both in the field and in the laboratory. These experiments, as any other economic experiments, have attempted to test theory and to design incentives to overcome common-pool resource dilemmas. Overall, the conclusions of these studies can be summarized as follows: (1) non-cooperative game theoretical solutions do not always hold; and (2) certain institutional settings increase cooperation levels.

A careful analysis of the relevant literature suggests that the focus of CPRE research has changed considerably over the last years. A first generation of studies aimed at contesting the non-cooperative game theoretical normative solution [2–4], whereas more recent works focused on policy implications in a myriad of different communities [5–9]. In 2011, [10] affirmed the need for yet another direction for CPREs, this time concerning “attributes of individuals as well as the social and social-ecological context in which they interact” in order to gain additional insights on the reasons behind users’ harvesting decisions. Most experimental designs of field studies account for the specific social settings of the system under investigation, but its ecological characteristics are often poorly represented. Thus, the time-continuous interaction between user behaviour and resource dynamics is not accounted for in a realistic manner. The consequences of this neglect for the external validity of the experimental results are unclear [11]. [10] suggested that a new wave of experiments should head towards intercultural comparisons, an aspect very much studied in experimental economics [12–16], and should especially address the ecological context of a social-ecological system.

However, moving towards intercultural comparisons omits aspects of how users, e.g. fishermen, decide on the quantity of their harvest [17]. Instead, CPREs keep centring their attention on institutional rules that determine “where, when, and how” one can harvest, and not on “how much” [17]. By contrast, research of exploited CPRs in natural sciences typically adopts a complementary perspective. When considering the case of fisheries, for example, arguably the CPR system of major global importance, the main research objectives have always focused on ecological aspects in order to minimize uncertainties in the assessment of exploited stocks and to ultimately optimize economic returns by adjusting extraction levels. However, although sophisticated methods have been successfully implemented to deal with the uncertainties involved, fishery management has all too often proven to be myopic, stretching cautionary scientific recommendations too far [18, 19], with consequences that have been at times catastrophic [20]. The more the quality of stock assessments and ecological models improved, however, the more apparent it became that human behaviour constitutes a considerable source of uncertainty. Even the most accurate stock assessment is of limited value when fishermen’s response to management cannot be anticipated. While this finding is neither new nor surprising [21, 22], the lack of understanding of human harvest behaviour is still regarded as a key problem in fishery management [23]. There is no simple and robust way to adequately integrate human behaviour into ecological resource management because it is context-specific and it continuously adapts to a changing environment, revealing all the characteristics of a complex adaptive system [24, 25].

Therefore, we argue here that a new generation of CPREs should incorporate attributes and characteristics of resource dynamics as well as elements of human behaviour that allow for an understanding of the time-continuous nature of effort choices and extraction levels. Incorporating the aspects just outlined in the design of CPREs will provide: (1) a much-needed push towards higher applicability of CPREs, (2) more relevance for urgent real-world problems, and (3) the capacity to produce more accurate policy recommendations for achieving a sustainable use of ecological resources in the long run.

Following these lines, we developed a new software system called OGUMI (https://www.ogumi.de), a powerful and robust yet flexible and easy-to-use tool (for example, likewise other existing software, it does not require expertise in behavioural game theory) for running CPREs in continuous time. In alternative to similar existing tools [26], which focus on standard game theoretical situations [27], are difficult to run in the field [28], or are not designed to capture real-time user responses to dynamically varying resources [29, 30], OGUMI produces high-frequency time-series chronicling the instantaneous adaptive reaction of humans to changing resource levels on mobile platforms.

OGUMI can be run on desktops, laptops, tablets, and mobile telephones. This offers the possibility to conduct experiments over telephone networks with thousands of participants. A tool that can be run on mobile devices presents several advantages [29]. Field experiments, which are an important complement to laboratory experiments, because they provide external validity and allow the use of a subject pool other than students, can be easily conducted in remote locations without the need to carry heavy equipments. Furthermore, computing devices have changed dramatically in the last years and usage has shifted from desktop computers to tablets and telephones, thus calling for platform-independent software [29].

OGUMI has been already used with over a hundred participants in actual harvest experiments in the laboratories of the Leibniz Centre for Tropical Marine Research, Bremen, Germany (results presented here in the section Testing OGUMI) and in the field in Mbour, Senegal, with local fishermen (manuscript in preparation).

OGUMI is open-source so that it can be used, modified and redistributed freely with the aims of fostering reproducibility and encouraging the undertake of field and laboratory experiments under dynamic changing conditions (e.g. under varying resource growth rate and effort levels). OGUMI also allows for conducting incentivised tasks and surveys.

## Materials and methods

### The system OGUMI

**Fig 1:**
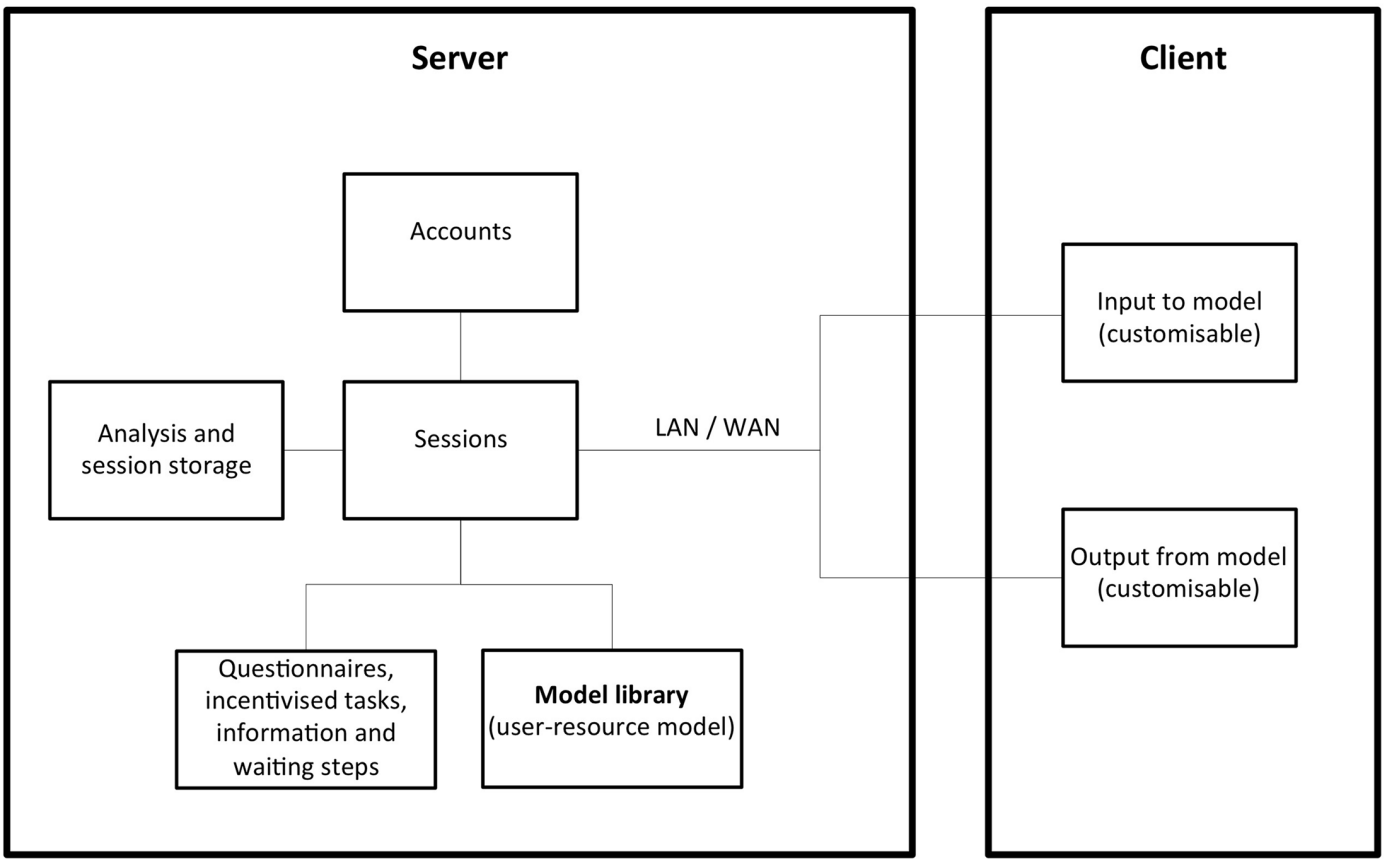
Schematic of OGUMI showing the major components of the system.

OGUMI is designed to capture varying effort levels in continuous time under changing ecological conditions. OGUMI is a free software: it can be redistributed or modified under the terms of the Apache License 2.0. The software includes three main components: a model library, a server, and a client. The interaction among the parts is qualitatively illustrated in Fig 1. Users interact with the system via a client interface, while administrators can design experiments and determine a number of standard options via a web-based interface. Under the current version, modifications on the client design or on the underlying mathematical model are implemented directly in the source code, which is written in Java. In the following, we describe the technical design of the system and elaborate on the two user interfaces and the standard model of OGUMI. The data of the pilot experiments described in the section Testing OGUMI were collected and analysed anonymously. More specific information about OGUMI is included in the official documentation of the software at https://www.ogumi.de.

### Model library

The library contains an abstract model class, which is the base of the user-resource model. Modification of the user-resource model should be consistent with the abstract model class.

The source code contains annotations that define input and output fields for the client (e.g. axes labels) and the numerical integrator of the ordinary differential equations constituting the user-resource model. An experiment is implemented in the abstract model class, the server recognizes the annotations, sends data necessary for displaying information to the client, and runs the user-resource model with the specified parameters.

All data from an experiment is saved on the server and can be downloaded at the end of the session as a CSV file.

### The standard user-resource model

The user-resource model that runs in the background is the classic Schaefer model [31, 32]. In spite of its simplicity, this model has proven very powerful for studying exploited fish stocks. A logistic term describes the growth of the resource *R*, while the harvest is a bi-linear function of the resource level and the cumulative effort ∑*E* that *N* users invest in the harvest. Hence,

$$\begin{array}{ccc} \frac{dR}{dt}\; & = & \;\mu_{R}R\left(1-\frac{R}{K}\right)-qR\sum_{i=0}^{N}E_{i} \end{array}$$ (1)

with *K* representing the carrying capacity, *μ*_*N*_ representing the maximum resource growth rate, and *q* representing the catchability per unit effort and resource unit.

The resource productivity is highest at *K*/2, while the harvest scales linearly with both effort and resource levels. The maximum sustainable yield, *MSY*, is achieved at

$$\begin{array}{c} E_{MSY}=\sum_{i=0}^{N}E_{i}\;=\;\frac{\mu_{R}}{2q}\; \end{array}$$ (2)

Assuming a specific cost *c* per unit of effort and a price *p* per resource unit, the return for an individual user *i* is given by

$$\begin{array}{ccc} B_{i}\; & = & \;pH_{i}-cE_{i} \end{array}$$ (3)

with the individual harvest *H*_*i*_ = *qRE*_*i*_.

Individual efforts of all users *E*_*i*_ are summed up and Eq 1 is then integrated forward in time with the cumulative effort of all users. The calculated future dynamics are valid as long as the cumulative effort remains unchanged. Whenever a user alters the effort *E*_*i*_, a new integration is carried out from that time with the updated ∑*E*_*i*_. While at constant effort the Schaefer model approaches a steady-state in *R*, repeated user intervention typically perturbs the dynamics of the system and displaces it from equilibrium.

Although this version of OGUMI is based on the Schaefer model, the system is flexible enough to accept any other user-resource model or it can be easily modified to include a multiple-species resource to study the effects of human extraction behaviour on spiecies diversity.

### The server

The server takes the experiment implementations and recognises input and output fields. It collects user inputs from the clients, forward them to the user-resource model and runs it. The communication between server and clients is realised using http and web sockets and all data is transferred via JSON, the JavaScript Object Notation. The system is thus flexible with respect to the client, can accept any other client that is able to communicate with a server, and can bring together remote participants via intranet or internet. Experiments with this system may be run for much longer time than typical laboratory-based set-ups, because the mobile app allows participants to follow an experiment even during their own daily activities. Besides the actual CPR experiment, a session may also comprise other stages such as Questionnaires, Incentivised Tasks, and Information, in arbitrary order. Finally, the server keeps records of all user inputs and model outputs in a database.

### The administrator interface

**Fig 2:**
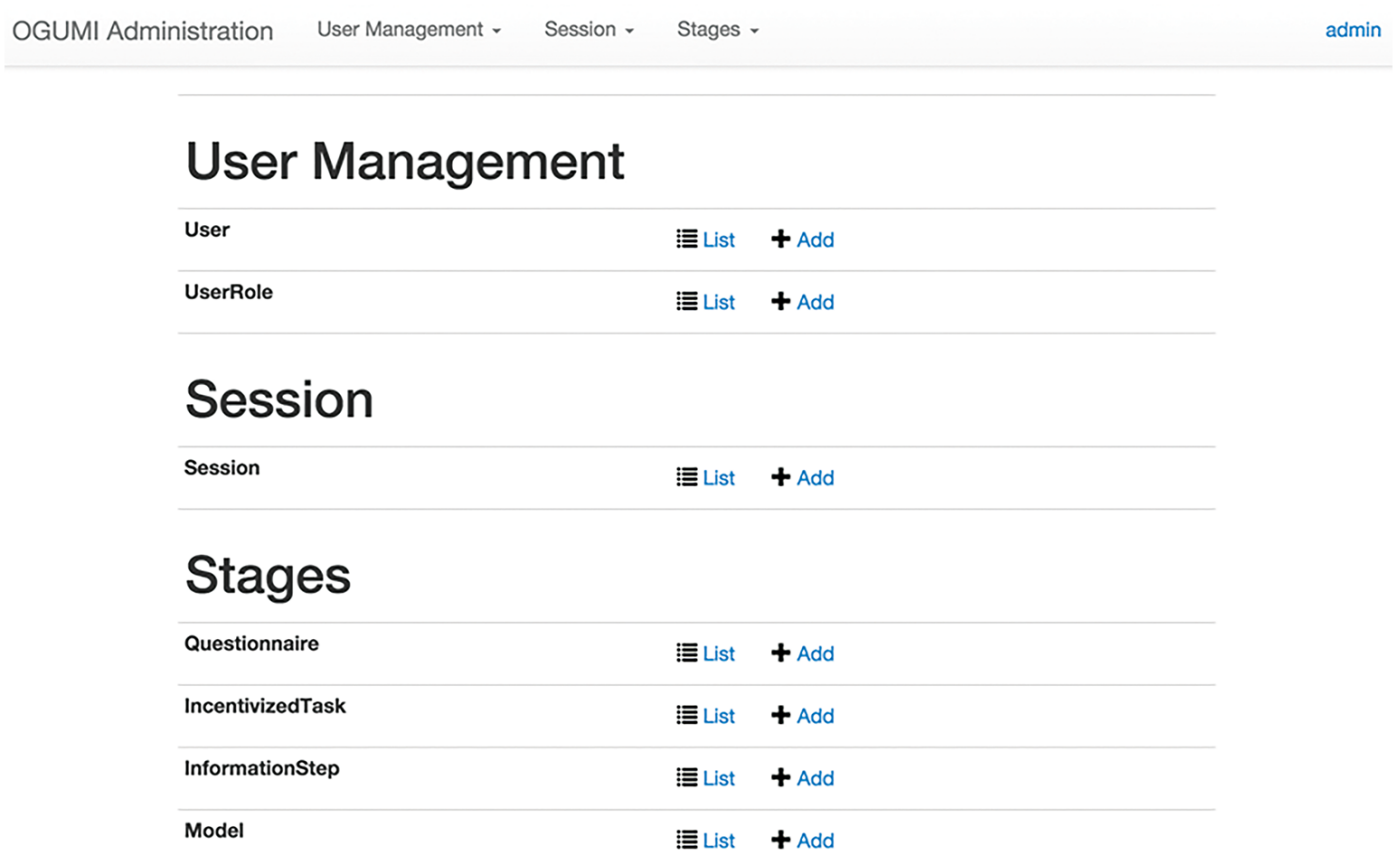
Main page of the administration interface, where users and stages are managed and experimental sessions are set-up.

The Administration System is a web interface that enables administrators to set-up, manage, and supervise experimental sessions without any programming skills. It is divided into three main sections named User Management, Session, and Stages (see Fig 2). The User Management menu contains two entries, User and User Role. The User menu lists all users, both active and inactive. New users can be added by the administrator or, alternatively, by any other user via the registration page in the client interface (see below).

The Stages menu lists the different types of available stages, i.e. Questionnaire, Incentivised Task, Information, and Model. In the respective sub-menus, new instances of these stages can be created or existing stages can be modified.

- *Questionnaire*: Consists of an arbitrary number of freely editable questions. Answers by users are collected via text fields, which can be restricted to numeric or text inputs.
- *Icentivized Task*: A choice experiment where the participant has to decide between two different allocations. For example, we implemented the distributional preferences task suggested by [33]. In this task participants are randomly paired and perform the same task, but remain anonymous to each other. The task consists in deciding how to allocate an amount of money between oneself—the active decision maker—and another participant—the passive decision maker. At the end of the stage, only one decision of both participants is randomly selected and actually paid out, i.e. the active and passive payments of the selected allocations are assigned to the respective participants. Another incentivised task that could be implemented in OGUMI is the risk aversion developed by [34].
- *Information Step*: These are steps that consist of an editable text field and an optional media element, which may include images, and video files. Information steps are typically used to present instructions of following tasks.
- *Model*: Are are listed all the available models and their corresponding files. In our example we would then select the Schaefer model. Models have to be compiled and packaged as .jar files. The model translations can be uploaded as .json, which can be downloaded from this menu. The model set-up including all the experiment-specific parameter values is determined in the Session menu (see below).

**Fig 3:**
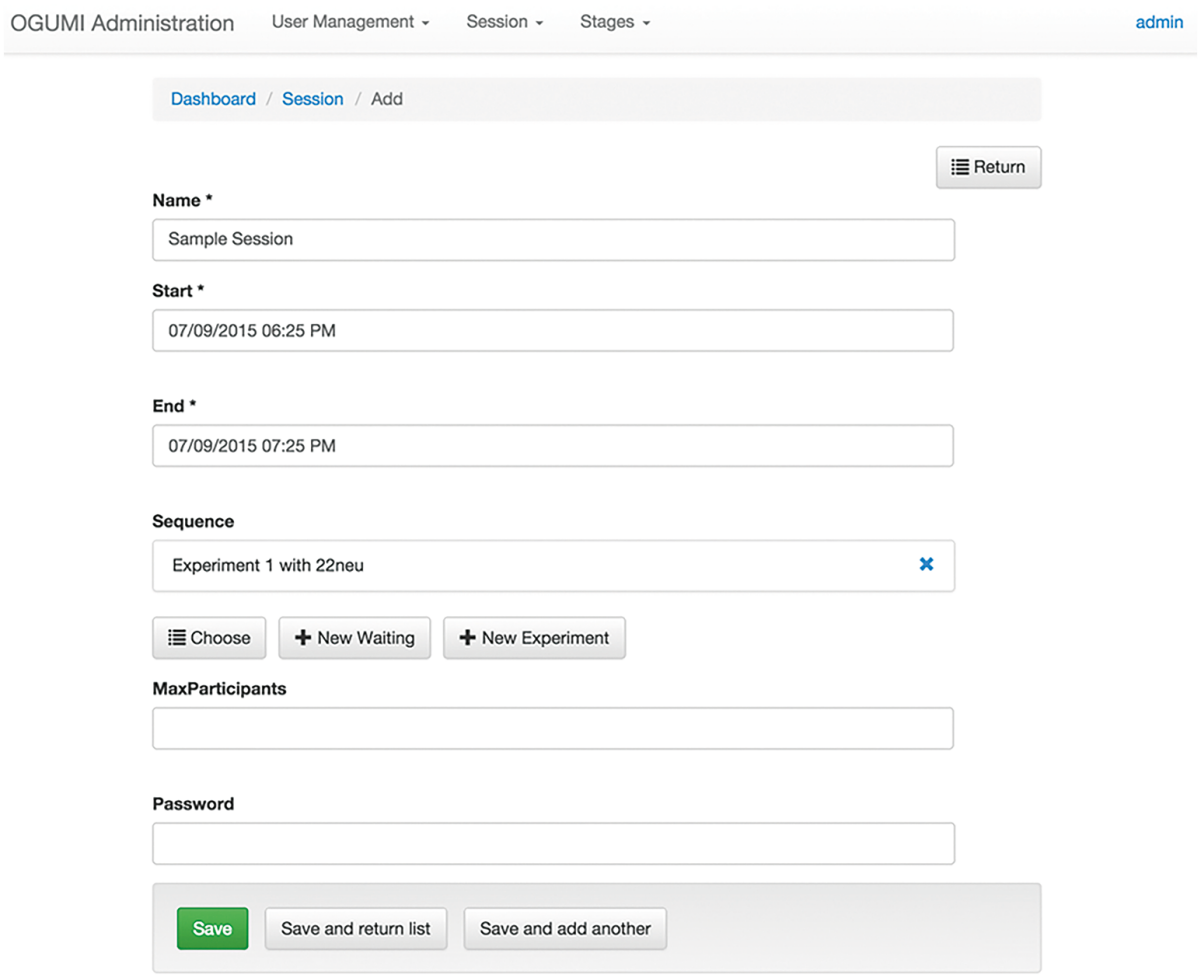
Creating a new session via the administrator interface. In this example, the session contains one experimental stage named ‘Experiment 1’, which uses the model ‘22neu’.

In the Session menu, new sessions can be created and existing sessions—completed, active, and upcoming—are listed. A session has fixed start and end times and contains an arbitrary sequence of stages and waiting steps, the latter representing freely definable waiting times between stages. Besides model experiments other stages can be added from the list of available stages (Fig 3).

**Fig 4:**
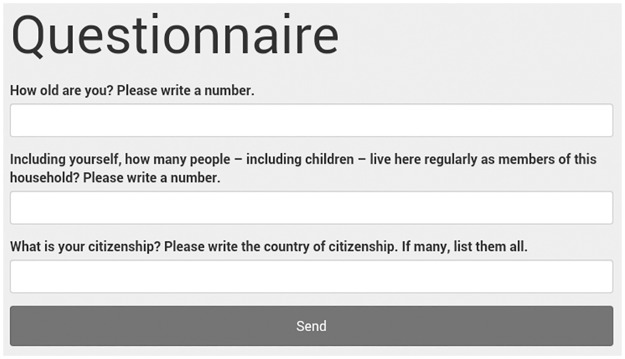
Live view of a running session in the administrator interface. The dynamic graph indicates the temporal evolution of the resource (orange) and the harvest of all users. In this example, users started harvesting at time *t* = 480 *s* and thereafter reduced the resource from its carrying capacity of 100 to almost 0 within 200 s. The administrator can also zoom to select the desired data range in the stylized graph on top of the main panel.

During an active session, a live view of the system provides basic monitoring. Active users are listed and the dynamics of the resource and the potential future trajectory of the resource are displayed in a graph. At the end of a session, the results of all stages can be downloaded as a compressed file. In addition, a figure showing the time-series of the resource and individual harvests is available for a visual check (Fig 4).

### The client

All interactions of users occur via the user interface on the client, which is an application for the mobile operating system Android 4.4 (or higher). Alternatively, OGUMI can also be run in web browsers. An experiment is organized within a session. A session consists of a linear sequence of different stages starting by default with user registration and log in. Users have to successfully complete one stage to be able to enter the next.

To participate in a session, a user has to log in with a registered user name and password. After log in, a user enters the Session stage by choosing an active session from a drop-down menu.

The sequence of the following stages is flexible and reflects the session design created by the administrator. As described above, a session may contain an arbitrary combination of waiting steps and at least one of the four different stages (Information, Questionnaire, Incentivised Task, and Model). Stages can be used more than once in a session and there is no limit for the number of total stages within a session.

**Fig 5:**
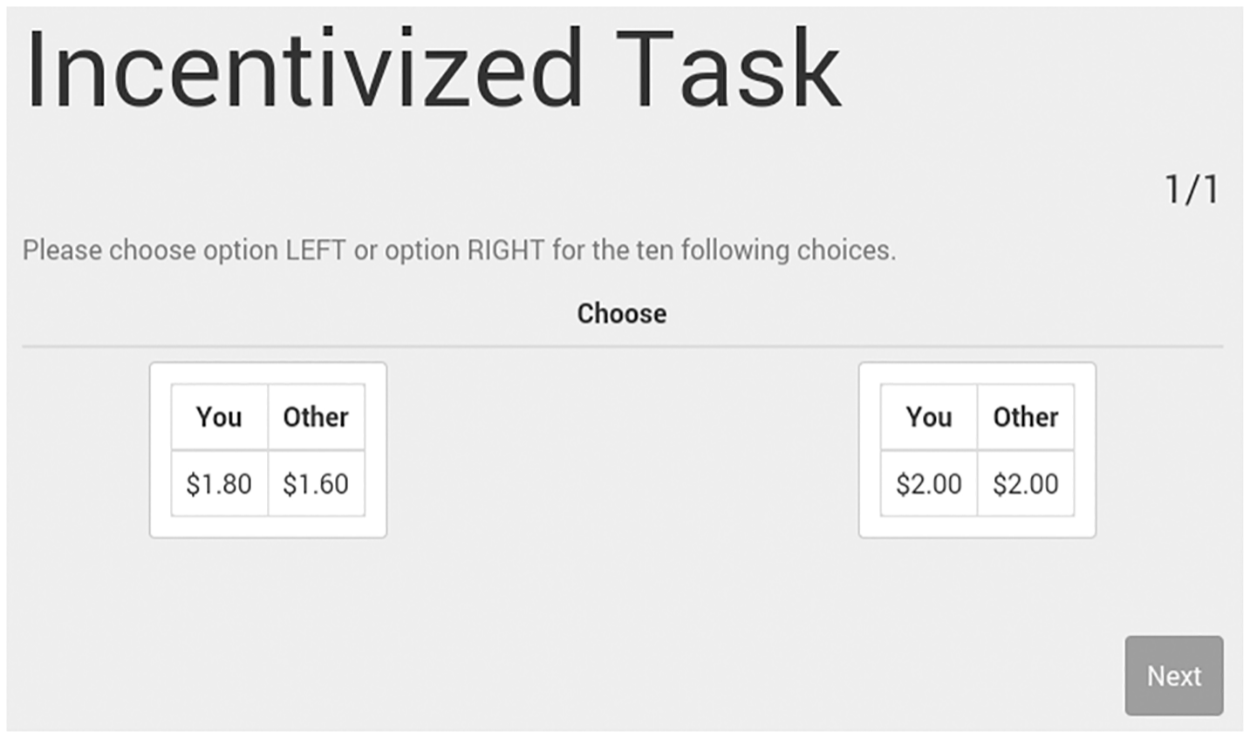
Client interface in an experimental stage. The coloured lines in the dynamic graph indicate the temporal evolution of the common pool resource (green) and the catch of the individual user (orange), whereas the numbers on the right of the graph show the cumulative catches of the individual user and of the entire group. The slider below the graph allows users to select the effort. Changes in effort have to be submitted by pressing the ‘Send’ button.

Different from all other stages, a Model experiment starts simultaneously for all users. Therefore, there is typically a waiting time before an experiment starts and a message informs the users about this. Once it starts, the experiment screen appears. It consists of three main elements: (1) a dynamic graph, (2) a slider with a send button, and (3) an area where several figures can be displayed (see Fig 5). In the graph, several time-dependent variables can be displayed, including the resource level and the individual harvest rate. The two y-axes can be scaled differently to increase readability. Depending on the model implemented, besides the individual harvest of the respective user—default figure—the average or the total harvest of all users can be shown. Instead of the harvest in resource units, it is possible to show the return in a desired currency by setting the price parameter accordingly (cf. Eq 3). In addition to the visual representation of the system’s dynamics, the numeric values for the cumulative and average harvest (or return) provide continuously updated information about the integrated outcome of the experiment. The administrator can freely choose which variables to show. A user interacts with the CPR and other users exclusively through the effort slider on the bottom of the screen by dragging the slider to the desired level. The change in effort will only be effective, i.e. submitted to the server, after the send button is pressed. The button will then be inactive for a few seconds, the time needed to transmit data, re-calculate system dynamics, and update all clients. When the experiment is over, a screen informs the participants about the amount they earned in the session.

**Fig 6:**
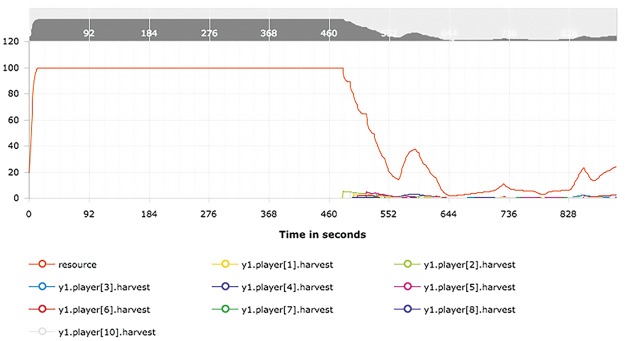
Screenshot of the questionnaire session.

**Fig 7:**
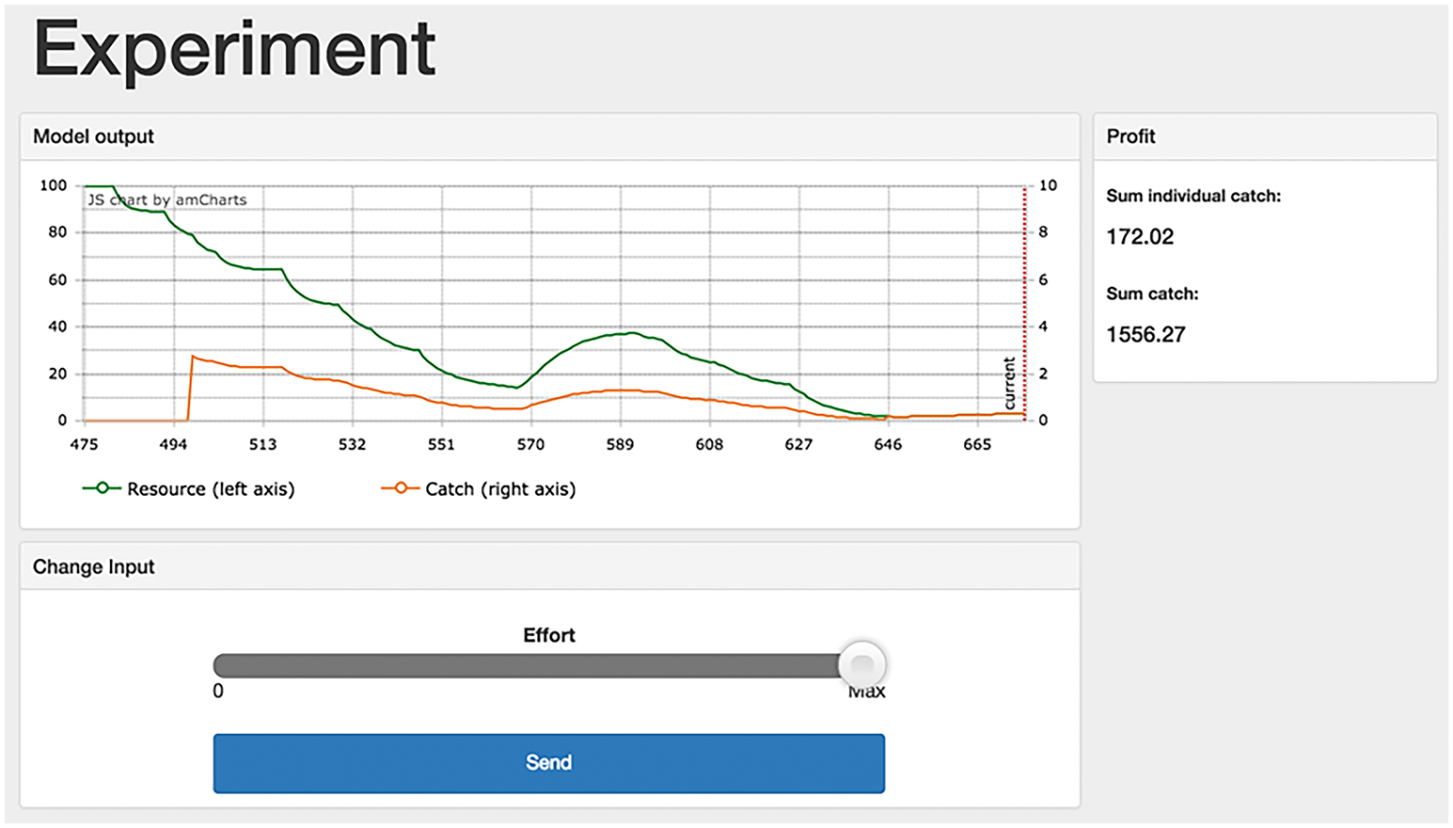
Screenshot of the incentivized task session.

Questionnaires and Incentivised Tasks are realised by text boxes and selectable buttons, respectively (see Figs 6 and 7).

Answers and choices are submitted by clicking a ‘next’ button. At the end of the Incentivised Task, the participants are redirected to a screen where they are asked to wait until their matching partner finishes the task. Before jumping onto the next part of the session, or finishing the session, participants are informed about their earnings from the Incentivised Task.

## Testing OGUMI

As explained, OGUMI is specifically designed to address and combine continuous-time, resource-user dynamics, characteristics of the resource, and large and heterogeneous sample sizes. To test the data generated by the software, we conducted a pilot experiment that mimicked a fishery.

A total of 72 participants from Bremen (Germany) participated in the pilot CPRE in August 2015. The experiment was conducted with Android tablets. All data were collected and analysed anonymously. Subjects were not allowed to participate in more than one session. After the CPRE, participants were asked to complete a questionnaire and perform an incentivised task. At the end, participants were presented with their final payoff, which was calculated as the sum of (1) earnings from the CPRE (2) 2 € for the questionnaire, and (3) one option of the incentivised task that was chosen at random. We run a total of 6 sessions during which the participants earned an average of 15.75 €.

Fig 5 shows the client interface during one of the sessions. Users rapidly reduced the CPR from the carrying capacity to almost zero 4. The individual catch of the user also decreased. Typical of CPR experiments in continuous time is the short pause of the initially linear decrease of the CPR, which in our case occurred between time steps 570 and 590. Users seemed to realise that harvesting at current pace would lead to a collapse and thus reduce their efforts. The CPR then recovered for a short period of time until the temptation of resuming intense harvesting prevailed again. Eventually, resource level and catches collapsed and remained at very low levels. Users hence failed to coordinate the collective task of sustainable harvesting from the CPR.

Major findings based on CPREs can be summarized as follows: when facing a social dilemma [4], aspects that build trust among users, such as communication [35, 36], sanctions [6, 37], and monitoring [38, 39], increase individuals’ cooperation levels. To prove that the data generated by OGUMI are robust and consistent with this findings, we explored the impact of communication on the resource level by running two different treatments:

1. Standard CPR experiment without face-to-face communication
2. Standard CPR experiment with face-to-face communication

**Fig 8:**
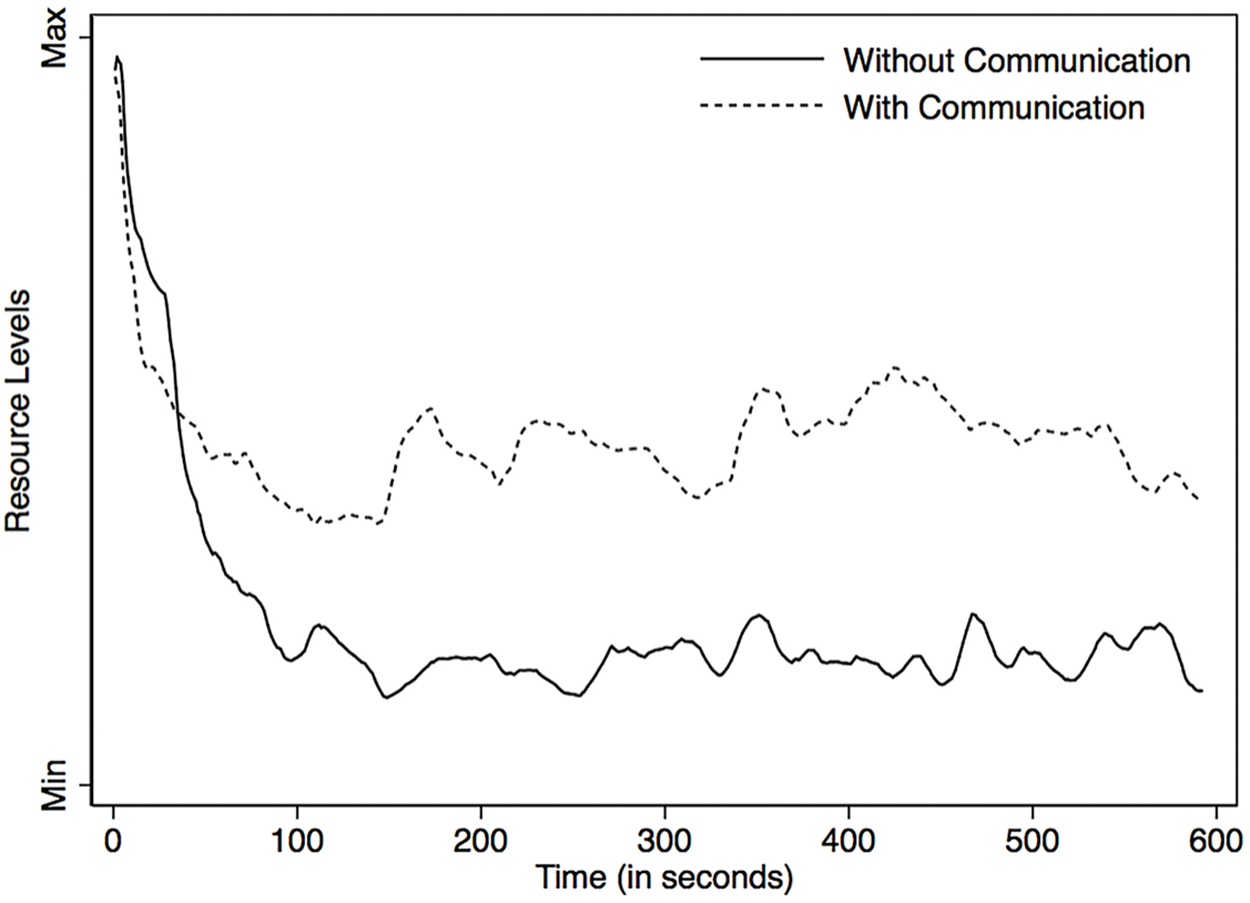
Temporal dynamics of resource levels obtained in each treatment.

**Fig 9:**
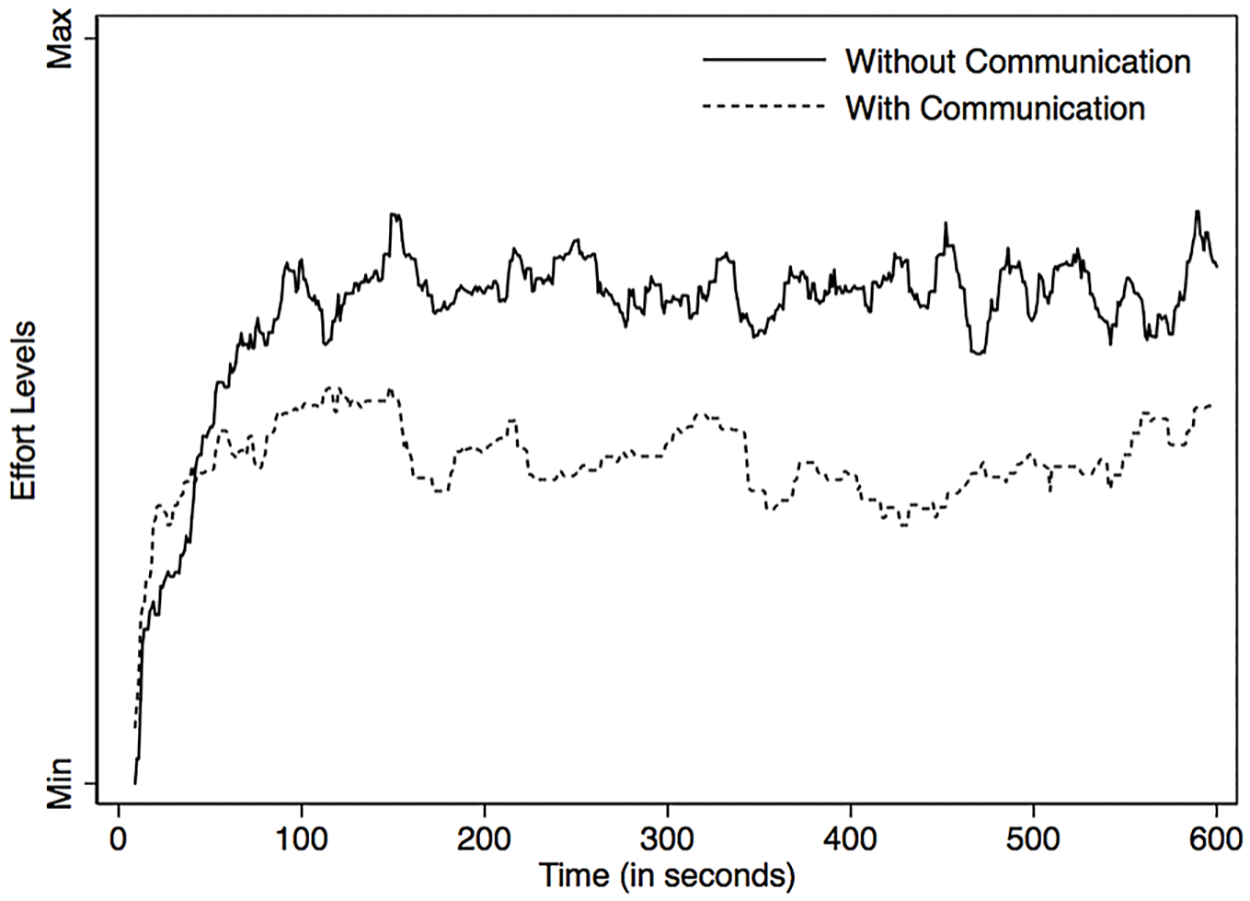
Temporal dynamics of effort levels obtained in each treatment.

The results of this test (Figs 8 and 9) reveal that the effort levels, which in OGUMI can be modified by users during the experiment, change consistently with the resource: higher effort levels are associated to lower resource levels. Resource levels (Fig 8) show that (1) both treatments follow the same trend and present an abrupt decline around time 100, after which the trend stabilizes, (2) the linear representation corresponding to the treatment without communication stabilizes to resource levels that are lower than those reached in the treatment with communication, and (3) both treatments exhibit similar volatility. From Fig 9 we observe that the trends are inverted compared to the resource levels: (1) both treatments follow the same trend and present an abrupt increase around time 100, after which the trend stabilizes; (2) the linear representation corresponding to the treatment without communication stabilizes to resource levels that are higher than those reached in the treatment with communication. However, in the treatment with communication, effort levels are less volatile than in the treatment without communication. This suggests that participants do not significantly deviate from their chosen collective exploitation strategy when choosing their effort levels.

These results are consistent with knowledge consolidated by many CPR experiments run in the field and in the laboratory: face-to-face communication encourages choices towards a more efficient outcome.

## Conclusions

Pen-and-paper methods have been for a long time the first choice for conducting CPREs. In the last years, however, computer-based experiments are becoming more popular. Several software systems have been developed [26] with z-Tree [30] being probably one of the most used. Recently, [40] made a significant step forward by abandoning the round-based design and instead adopting a time-continuous and spatially explicit five-player lab-experiment. Several authors have begun to explore human behaviour with continuous-time economic experiments using customised software [27, 40–45]. An essential finding of these works is that the possibility of participants to instantaneously react to ecological changes substantially increases cooperation. This suggests that experimental designs adopting discrete time analysis could be producing misleading environmental and conservation public policy suggestions, especially in the current context of abrupt environmental changes and rapid degradation of ecosystem services.

In relation to existing tools, OGUMI presents the following new features. 1) A large number of users can interact by harvesting from a dynamically changing ecological resource (in its current setup OGUMI mimics fisheries); 2) Users can respond to ecological changes in real-time by altering their efforts; 3) it is flexible as it can be (a) customized to account for different components of the ecosystem under study, (b) framed for different resources and multiple species thus accounting for biodiversity changes, and (c) configured to capture perturbations and shocks in resource abundance; 4) it produces high-frequency time-series chronicling the instantaneous adaptive reaction of users to current resource levels; 5) it is not a tool exclusive for the behavioural economist, i.e. it does not require expert knowledge on standard economic games, and can be easily used by ecologists interested on experimenting with the dynamical aspects of human-resource interactions.

We provide OGUMI as free software under the Apache License 2.0 (http://www.apache.org/licenses/). The source code, for both client and server, and full documentation are available in GitHUB (https://github.com/ogumi), a web repository that offers distributed version control and source code management functionalities such as bug tracking. A compiled, ready-to-use version of OGUMI, which we use to run the experiments presented in this paper, and the necessary documentation are available at https://www.ogumi.de. We ask that people cite the present paper when using OGUMI for academic or other purposes.
